# Impact of Mannose-Binding Lectin Deficiency on Radiocontrast-Induced Renal Dysfunction

**DOI:** 10.1155/2013/962695

**Published:** 2013-12-10

**Authors:** Michael Osthoff, Marten Trendelenburg

**Affiliations:** ^1^Department of Infectious Diseases, University Hospital Basel, Petersgraben 4, 4031 Basel, Switzerland; ^2^Laboratory of Clinical Immunology, Department of Biomedicine, University Hospital Basel, Petersgraben 4, 4031 Basel, Switzerland; ^3^Clinic for Internal Medicine, University Hospital Basel, Petersgraben 4, 4031 Basel, Switzerland

## Abstract

Contrast-induced nephropathy (CIN) is the third leading cause of acute renal failure in hospitalized patients. Endothelial dysfunction, renal medullary ischemia, and tubular toxicity are regarded as the most important factors in the pathogenesis of CIN. Mannose-binding lectin (MBL), a pattern recognition protein of the lectin pathway of complement, has been found to aggravate and mediate tissue damage during experimental renal ischemia/reperfusion (I/R) injury which was alleviated by inhibition with C1 inhibitor, a potent MBL, and lectin pathway inhibitor. In this paper, we highlight the potential role of MBL in the pathogenesis of human CIN. In experimental I/R models, MBL was previously found to induce tubular cell death independent of the complement system. In addition, after binding to vascular endothelial cells, MBL and its associated serine proteases were able to trigger a proinflammatory reaction and contribute to endothelial dysfunction. In humans, urinary MBL was increased after administration of contrast media and in individuals with CIN. Moreover, individuals with normal/high MBL levels were at increased risk to develop radiocontrast-induced renal dysfunction. Hence, MBL and the lectin pathway seem to be a promising target given that a licensed, powerful, human recombinant inhibitor exits to be added to the scarce armamentarium currently available for prophylaxis of CIN.

## 1. Introduction

Iodinated contrast media (CM) are an essential component of contemporary imaging and interventional studies, and its use is steadily increasing as a consequence of the exponential growth of contrast studies over the past decade [1]. Although CM are generally well tolerated, they have been causally linked to acute kidney injury known as contrast-induced nephropathy (CIN). CIN has become the third leading cause of acute kidney injury in hospitalized patients after impaired renal perfusion and nephrotoxic medication accounting for approximately 10% of cases [2]. Consequently, this iatrogenic complication is associated with extended length of stay, accelerated onset of end-stage renal disease, need for dialysis, 4-fold increased short and long-term mortality [3], and increased health care costs compared to patients who do not develop CIN [4, 5]. Preexisting renal impairment, diabetes mellitus, advanced age, congestive heart failure, simultaneous use of nephrotoxic drugs, hypovolemia or large volumes, and repeated use of CM have been previously identified as risk factors for CIN [6]. For research purposes, a rise in serum creatinine concentration of more than 25% or 44.2 *μ*mol/L (0.5 mg/dL) within 3 days of intravascular administration of CM has been arbitrarily chosen to diagnose CIN in the absence of an alternative cause. Despite numerous attempts, preventive strategies are largely confined to intensive hydration with sodium chloride [7] and potentially the use of sodium bicarbonate [8], which might be related to the complex pathophysiology of CIN with the exact mechanisms yet to be elucidated [9].

## 2. Pathophysiology of Contrast-Induced Nephropathy

**Figure 1:**
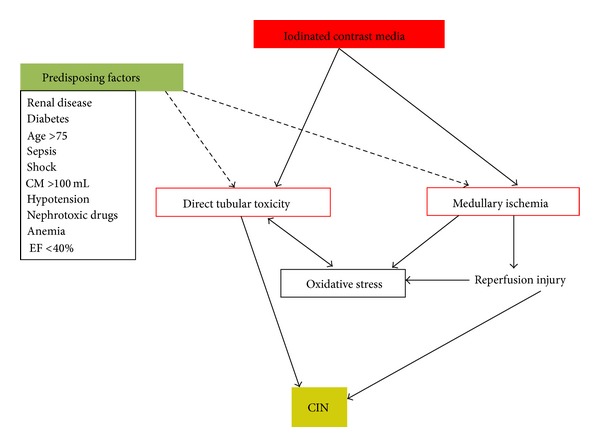
Pathogenesis of contrast-induced nephropathy. Abbreviations: CM, contrast media; EF, ejection fraction; CIN, contrast-induced nephropathy.

Evidence from numerous studies suggests that a combination of several mechanisms is responsible for the development of CIN [10, 11] including direct renal tubular and endothelial cell cytotoxicity, regional ischemia/reperfusion injury, and increased viscosity-induced renal damage (Figure 1). Local renal ischemia is a direct result of CM induced prolonged vasoconstriction, which primarily affects the descending vasa recta of the outer medulla. Recent data indicate that a direct cytotoxic effect of CM on endothelial cells with subsequent endothelial cells dysfunction is primarily responsible for the vasoconstriction in the outer medulla [12]. Hypoxic injury to this region is aggravated by an increased tubular cell oxygen demand after administration of CM. Consequently, oxidative stress, which enhances the production of reactive oxygen species (ROS) and triggers a local inflammatory response, may cause additional cell injury during the reperfusion phase which follows the initial tissue ischemia [13]. The second important effect of CM involves direct cytotoxic damage to renal tubular cells which seems to be mediated by interference with mitochondrial enzyme activities leading to the generation of ROS, apoptosis of tubular cells ultimately contributing to acute tubular injury [14].

In this paper, we will discuss mannose-binding lectin (MBL), a complement protein which has been implicated in apoptosis and ischemia/reperfusion (IR) injury in various organs, as a mediator/aggravator of contrast-induced local renal damage.

## 3. Mannose-Binding Lectin

**Figure 2:**
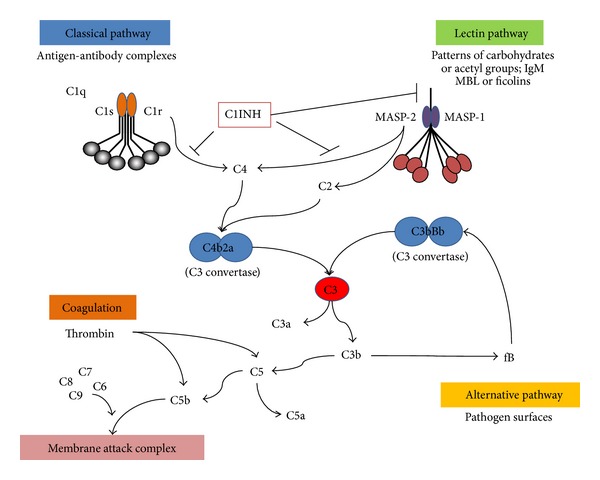
Schematic representation of the complement cascade and its three pathways. Each of these pathways is triggered by different molecules on pathogen or foreign/dying cell surfaces. These three pathways merge at the level of the C3 convertase subsequently giving rise to the same effector molecules. Recent data indicate that the coagulation cascade is linked with the complement system via thrombin which acts as C5 convertase. Abbreviations: C1INH, C1 inhibitor; MBL, mannose-binding lectin; MASP, mannose-binding lectin associated serine protease.

Mannose-binding lectin (MBL) is a circulating innate pattern-recognition protein of the complement system that is primarily synthesized in the liver and rarely detected in other organs in the absence of inflammation [15]. Its carbohydrate recognition domain binds to molecular patterns (consisting of certain sugars such as D-mannose and N-acetyl-D-glucosamine) on ligand surfaces in a calcium dependent manner including nonself (various bacteria and viruses) as well as endogenous epitopes (apoptotic and necrotic cells) [16, 17]. Subsequently, MBL-associated serine proteases-1 and -2 (MASP) are activated and cleave both C4 (MASP-2) and C2 (MASP-1 and -2) with consequent complement activation and opsonophagocytosis (Figure 2). Recently, a direct link between the MBL-MASP complex and the coagulation cascade has been shown without the involvement of downstream complement components [18, 19]. Several mutations in the *MBL2 *gene negatively influence the concentration of circulating functional MBL multimers [20]. The *MBL2* gene is located on chromosome 10q21.1 and at least 6 single nucleotide polymorphisms in the promoter and exon 1 regions segregate under linkage disequilibrium to produce 7 common haplotypes of MBL. In the literature, exon 1 variant alleles are often collectively designated as O and the wild-type gene as A, and the most influential promoter variant allele and the wild-type gene designated as X and Y, respectively [21]. As a consequence of exon 1 mutations, lower order oligomers lack the binding capacity and ability to activate the complement cascade. Beside genetics several environmental factors including thyroid function [22] and growth hormones [23] have been identified to directly influence the synthesis in the liver. In fact, serum levels can vary several folds in individuals with identical genotype. Serum MBL levels range from complete absence to 10,000 ng/mL in all populations tested to date, and low, intermediate, and high levels correlate to a great degree with low (O/O and O/XA), intermediate (XA/XA, YA/O), and high producing *MBL2* genotypes, respectively [24]. Overall, low producing MBL genotypes can be observed in up to 30% of the many populations tested to date with no functional multimer detectable in about 10% [25]. The significance of low or absent MBL levels has not finally been determined in healthy individuals. However, ample evidence suggests that MBL deficiency might negatively impact on the risk of serious infections when the adaptive immune system is either immature (e.g., in neonates [26, 27]) or severely compromised (e.g., after transplantation [28–30]).

More recently, MBL has been implicated in apoptosis and ischemia/reperfusion (IR) injury, two proposed main mechanisms in CIN [31].

There are scarce data on the role of MBL and the lectin pathway of complement in CIN, essentially limited to two human studies [32, 33] without any evidence from rodent or *in vitro *models. Hence, we will discuss the relevance of MBL and the lectin pathway in different pathophysiologic mechanisms implicated in CIN assuming analogy.

## 4. MBL and Ischemia/Reperfusion Injury

Local I/R is regarded as being at least in part responsible for the development of CIN. Conversely, plentiful rodent [34–37] and human studies [38, 39] have highlighted the crucial role of MBL in aggravating the inflammatory response and tissue damage during I/R injury of various organs including the kidneys. In a mouse model of renal ischemia and reperfusion, de Vries et al. [40] were the first to demonstrate involvement of MBL in renal I/R injury, that is, renal deposition of MBL which colocalized with late complement factors and correlated with complement activation, neutrophil influx, and organ damage, whereas activation of the classical pathway of complement was not detected. However, a positive staining for MBL was only observed after at least 30 minutes of renal ischemia. Of note, the duration of local hypoxia in CIN is unknown. In line with the animal data, glomerular MBL deposition was also observed early after kidney transplantation in non-heart-beating donor kidneys as compared to heart-beating donor kidneys (which do not suffer from prolonged warm ischemia) and in renal tissue from pretransplant biopsies, and peritubular and tubular MBL deposition in primary nonfunctioning as compared to delayed functioning kidney transplants. Further studies with MBL knock-out mice confirmed the crucial role of MBL in aggravating tissue damage as knock-out mice were protected from renal I/R injury with reestablishment of organ injury after reconstitution with recombinant MBL [41]. Complement activation was diminished in knock-out mice after I/R injury, and recombinant MBL was only deposited around proximal tubules in injured kidneys as compared to sham-operated kidneys indicating exposure of neoepitopes in the kidneys after I/R. In a swine model of renal I/R injury Castellano et al. [42] confirmed the principal involvement of the lectin pathway of complement as compared to the classical and alternative pathways. In addition, therapeutic inhibition with recombinant C1 inhibitor (which inhibits activation of the classical and lectin pathways) led to diminished complement deposition, influx of inflammatory cells and tubulointerstitial damage.

How does MBL inflict renal damage after I/R injury? While activation of the complement system after binding of MBL to hypoxic cells with subsequent killing and opsonophagocytosis of these cells might seem plausible, a recent study has shed light on a new mechanism independent of complement activation [43]. In a rat model of renal I/R inhibition of C5 (which results in complete inhibition of the terminal pathway of complement) and C3 did not reduce renal dysfunction as compared to inhibition of MBL indicating that activation of complement pathway downstream of C4 is not a crucial event early after reperfusion. Instead, MBL was shown to leak from the circulation into the renal interstitium immediately upon reperfusion with subsequent internalization by tubular cells followed by induction of cell death. These data suggest that MBL-mediated cell death precedes complement activation and seems to be the primary culprit of renal tubular injury after I/R. Interestingly in a recent study, MBL was strongly upregulated in the urine proteome profiles of individuals after application of CM, in particular in individuals that developed CIN [33]. As MBL expression is usually not detectable in human kidneys, it seems plausible that urinary MBL originated from the circulation and leaked into the kidneys as a result of CM induced renal ischemia and subsequent injury. This would indicate that the pathophysiology of CM-induced renal damage is comparable to experimental renal I/R injury, at least to a certain extent, with involvement of MBL and the lectin pathway in both scenarios. Indeed, results from a recent study indicate that CM induces renal tubular apoptosis via reactive oxygen species and the intrinsic apoptotic pathway [44], which could be mediated and/or amplified by MBL. However, a direct link between MBL and increased intracellular production of reactive oxygen species remains to be determined.

How does MBL bind to reperfused renal parenchymal cells? Circulating natural IgMs have long been implicated in reperfusion-mediated damage by binding to ischemia-conditioned tissue and activating the complement cascade as shown in mice totally deficient in immunoglobulins [45, 46] or complement receptor-2 [47]. Natural IgMs were found to bind to self-antigens that become exposed after ischemic conditioning [48], and IgM deposition on ischemic tissue was shown to precede complement activation during experimental skeletal muscle and intestinal I/R injury [37]. Indeed, several studies have revealed that both natural IgM and MBL are necessary for complement activation and subsequent tissue injury following ischemia and reperfusion [37, 49]. Consequently, MBL was also found capable of binding to IgM with subsequent activation of the lectin pathway of complement [50]. In agreement with data presented above in other organs, natural IgM deposited within the glomeruli was crucially involved in I/R injury of the kidneys without involvement of the classical pathway of complement [51]. Taken together, it seems that natural IgM bind to neoepitopes which are expressed in the postischemic kidney with subsequent binding of MBL to IgM followed by induction of apoptosis as demonstrated in renal tubular cells or immediate activation of the lectin pathway of complement with amplification via the alternative pathway [52], deposition of the membrane attack complex and recruitment of inflammatory cells as demonstrated in glomeruli.

## 5. MBL and Endothelial Cell Dysfunction

MBL does not only seem to aggravate reperfusion damage by impacting on renal parenchymal cells but also via its binding to endothelial cells. Over a decade ago, Collard et al. demonstrated binding of MBL to endothelial cells immediately after oxidative stress with subsequent endothelial C3 deposition, which could be prevented by anti-MBL monoclonal antibodies [53]. Further studies lead to the identification of cytokeratin 1 as a mediator forMBL deposition and thus auto-antigens presented on hypoxic vascular endothelial cells causing local activation of the complement system via the lectin pathway [54]. *In vivo*, MBL was found to colocalize with IgM and complement C3 on the endothelial surface of ischemic brain vessels but not in vessels of non-ischemic ipsilateral brain territories or the contralateral hemisphere [55]. Similar data is not available in renal I/R models. However, MBL was found to be deposited on tubular cells and in peritubular capillaries in colocalization with complement C6 in mice [40] and complement C4d in pigs [42]. Independent of downstream complement activation binding of MBL to stressed endothelial cells might also impact on platelet activation and amplification of the coagulation cascade. Indeed, MBL-associated serine protease-1 (MASP-1) induced activation of protease-activated receptor 4 (PAR4) in human umbilical vein endothelial cells [56]. Of note, endothelial cells can be activated via PAR4 (by thrombin) in the setting of vascular damage [57] which in turn might contribute to the regulation of thrombotic events, inflammation, and vascular permeability. Hence, MBL binding to endothelial cells might not only activate the complement system but also enable contact of MASP-1 with endothelial cells in order to trigger proinflammatory reactions by cleaving PAR4. Further support for this hypothesis comes from recent studies demonstrating that MBL and MASP-1/MASP-2 are directly involved in clot formation *in vitro *[19, 58] and *in vivo *[18]. In fact, vascular endothelial cell injury led to MBL deposition along the vascular endothelium along with MASP-1 mediated cleavage of thrombin substrates, enhancement of platelet aggregation, and *in vivo* thrombogenesis [18]. Of note, these events were independent of downstream complement activation. In summary, MBL and the lectin pathway could contribute to vascular endothelial dysfunction, the primary driver of vasoconstriction in the outer medulla in CIN. In the sequence of events MBL might have an aggravating role in both the ischemia and the reperfusion phase of CIN with different targets in both phases. As outlined in this paragraph MBL could first impact on vasoconstriction and hence ischemia in the outer medulla in CIN. Subsequently, leaked MBL could bind to reperfused renal parenchymal cells in the reperfusion phase causing additional damage with CIN as the overall outcome.

## 6. MBL and Contrast-Induced Nephropathy

After reviewing the impact of MBL and the lectin pathway in renal I/R injury in general, we will discuss human studies that have examined this pathway in CIN. Wang et al. analyzed urine samples from 12 patients undergoing diagnostic or therapeutic cardiac catheterization with two-dimensional fluorescence differential gel electrophoresis followed by liquid chromatography mass spectrometry [33]. Among the proteins identified, MBL and MASP-2 were significantly upregulated in the urine samples taken 12–18 hours after administration of CM compared to the pre-procedural urine sample. In addition, urine levels of MBL increased at least two-fold in 13 patients who developed CIN after cardiac catheterization whereas urinary MBL levels remain stable in 18 non-CIN patients with similar baseline characteristics and procedures carried out. Whether renal cells are able to produce MBL during hypoxic stress is unknown, but MBL expression was not found in (presumably) unstressed renal tissue previously [15]. Hence, it is reasonable to hypothesize that MBL might have leaked from the circulation into the hypoxic/stressed area in the kidneys in response to regional ischemia and vascular dysfunction rather than being secreted by renal tubular cells. Given the advantage of MBL deficiency in rodent models of renal I/R injury, a second study tested the hypothesis that MBL deficiency in patients undergoing contrast studies would be associated with a reduced incidence of CIN [32]. Baseline serum MBL levels were analyzed in 246 patients with pre-existing renal dysfunction (median (IQR) eGFR 44 (35–52) mL/min/1.73 m^2^) who were part of a randomized controlled trial examining three different prophylactic regimens. Interestingly, the incidence of CIN as defined by the standard definition (increase in serum creatinine concentration of more than 44 *μ*mol/L or 25% within 48 hours after exposure to CM) was only 6.5% in the whole cohort. MBL levels in patients who experienced an acute deterioration of renal function did not differ significantly from patients that showed stable creatinine concentrations. Similarly, the incidence of MBL deficiency (defined as MBL levels < 500 ng/mL) was nearly identical in the two groups. However, analysis of cystatin C kinetics, a more sensitive and rapid indicator of changes in GFR than serum creatinine [59, 60], revealed an association of low MBL levels with a reduced deterioration in renal function after CM administration. Indeed, individuals with MBL deficiency were two-times less likely to develop a significant cystatin C increase after contrast studies as compared to patients with MBL levels regarded as sufficient, and there was a trend towards a reduced length of stay in the former group. However, MBL deficiency was not associated with superior clinical outcomes in this study. In summary, high MBL levels might predispose to contrast-media induced renal dysfunction as MBL seems to be involved in the pathogenesis of CIN. Because of the limited evidence additional studies in patients at high risk for CIN are needed to fully elucidate the role of MBL in the pathogenesis of human CIN.

## 7. MBL and Prevention of CIN

In contrast to clinical scenarios of acute ischemia (myocardial infarction, ischemic stroke) where detrimental MBL binding and activation of the complement cascade has already occurred before pharmacological inhibition is feasible, CIN does offer the unique opportunity to attenuate renal injury by interfering with contributing pathways before they become activated. Indeed, treatment with anti-MBL monoclonal antibodies or inhibitors of MASP-1/-2 might be possible in advance of CM administration similar to hydration with sodium chloride, even in the setting of an acute ischemic event. Additionally, inhibition of MBL and the lectin pathway in the latter settings might even “kill two birds with one stone” by ameliorating not only CIN but also tissue damage caused by the initial ischemic event and subsequent reperfusion via therapeutic revascularization. However, clinical data supporting a role of MBL in CIN are still very limited, and there is clearly a need for further studies in humans.

Given the fact that renal injury after I/R seems to be mediated independently by MBL or via MASP-1/-2 and activation of the lectin pathway, pharmacological candidates should ideally interfere with MBL and the lectin pathway as far upstream as possible. Hence, antibodies against MBL or inhibition of MASP-1/-2 seem to be preferable compared to inhibition of C3 and C5 or even further downstream proteins. Indeed, transient inhibition of the lectin pathway by systemic pretreatment with inhibitory MASP-2-specific monoclonal antibodies was effective in significantly ameliorating gastrointestinal I/R in a rodent model [61]. Recombinant human C1 inhibitor (rhC1INH) is even more promising in our opinion. RhC1Inh is a multiple-action-multiple-target inhibitor that interferes with C1 of the classical pathway and to a lesser extent the alternative pathway of complement, the kinin and the coagulation system [62]. Of note, rhC1INH also binds to and inhibits MBL [63] and MASP-2 [64] and hence, activation of the lectin pathway by MBL or ficolins—a major advantage over plasma-derived C1 inhibitor [63]. RhC1INH showed very promising results in an animal model of transient cerebral ischemia when administered up to 18 hours after ischemic stroke [63] whereas plasma-derived C1 inhibitor and MASP-2 specific antibodies were mainly effective when administered before the acute ischemic event. Similarly, administration of rhC1INH led to significant reduction in complement deposition and significant inhibition of tubular damage and tubular epithelial cells apoptosis in a swine model of renal I/R injury [42]. Of note, this MBL inhibitor has already been approved for hereditary angioedema in Europe (rhC1INH, Ruconest) having demonstrated a very favorable side-effect profile. Nevertheless, caution should be emphasized when translating results from animal studies to human trials, as no single animal model can truly replicate CIN patients in all their complexity. However, given the above mentioned evidence, the paucity of available effective prophylactic treatment options in high-risk CIN patients and the lack of alternatives, in particular regarding intravascular interventions, human studies to explore the effectiveness of blocking MBL and the lectin pathway in CIN using rhC1INH are clearly desirable. Ideally, rhC1INH should be administered once during prehydration with normal saline before the contrast study, and patients with several risk factors for the development of CIN should be included. Similar to treatment of hereditary angioedema, a single treatment dose is not expected to cause major side effects including major infections.

## 8. Conclusion

CIN is still a frequent complication of diagnostic and interventional contrast studies and associated with significant morbidity and long-term mortality. After decades of research, prophylactic treatment is essentially still limited to hydration with normal saline and potentially the use of sodium bicarbonate, an unsatisfactory fact. Fortunately, over the last decade our knowledge about the development of CIN has steadily increased and led to the identification of I/R injury as the culprit event in the complex pathophysiology of CIN. Similarly, ample evidence suggests that MBL and the lectin pathway of complement aggravate the course of I/R in various organs including the kidneys. Hence, it seems plausible that MBL might also impact on the severity of CIN in patients at risk. Evidence from renal I/R injury models imply that the detrimental effects of MBL are propagated by induction of endothelial cell dysfunction, tubular cell apoptosis, activation of the complement cascade, and recruitment of neutrophils. Preliminary evidence from two human studies suggests that the harmful effects of contrast media are at least partly mediated by MBL and the lectin pathway. As effective inhibitors of MBL and the lectin pathway are already available for prophylactic treatment of CIN in humans, there is a need of additional animal and human studies to fully elucidate the role of MBL in the pathogenesis of CIN. In the context of a predicted further increase in diagnostic and interventional contrast studies and a still scarce choice of prophylactic treatment options, research regarding MBL and the lectin pathway in CIN seems to be promising in our opinion.
